# Quantification of Glycan in Glycoproteins via AUCAgent-Enhanced Analytical Ultracentrifugation

**DOI:** 10.3390/ph19020210

**Published:** 2026-01-26

**Authors:** Xiaojuan Yu, Zhaoxing Wang, Chengshi Zeng, Ruifeng Zhang, Qing Chang, Wendan Chu, Qinghua Ma, Ke Ma, Lan Wang, Chuanfei Yu, Wenqi Li

**Affiliations:** 1 State Key Laboratory of Drug Regulatory Science, NHC Key Laboratory of Research on Quality and Standardization of Biotech Products, NMPA Key Laboratory for Quality Research and Evaluation of Biological Products, National Institutes for Food and Drug Control, Beijing 102629, China; yuxiaojuan@nifdc.org.cn (X.Y.); wanglan@nifdc.org.cn (L.W.); 2 Beijing Frontier Research Center for Biological Structure, Tsinghua University, Beijing 100084, China; wangzhaoxing@mail.tsinghua.edu.cn (Z.W.); zengchengshi@mail.tsinghua.edu.cn (C.Z.); rui071312@mail.tsinghua.edu.cn (R.Z.); changqing@mail.tsinghua.edu.cn (Q.C.); chuwendan@mail.tsinghua.edu.cn (W.C.); 3 Core Facility for Biomolecule Preparation and Characterization, Technology Center for Protein Sciences, Tsinghua University, Beijing 100084, China; 4 Beijing Zhifei Lvzhu Biopharmaceutical Co., Ltd., No. 6, Taihe Third Street, Beijing Economic Development Zone, Beijing 100176, China; maqinghua@zhifeishengwu.com (Q.M.); make@zhifeishengwu.com (K.M.)

**Keywords:** glycoprotein, quantification, analytical ultracentrifugation, AUCAgent

## Abstract

**Background**: As essential biomolecules composed of proteins and carbohydrate moieties, glycoproteins play pivotal roles in numerous biological processes. The glycosylation level plays a crucial role in determining the functionality of glycoproteins. Therefore, the precise quantification of glycan components in proteins holds significant importance for research on and development of polysaccharide–protein-conjugated vaccines. **Methods**: In this study, a novel glycan quantification approach was developed, leveraging analytical ultracentrifugation (AUC) technology that synergistically utilizes ultraviolet wavelength absorption and interference data to directly determine glycan mass fractions in glycoproteins. **Results**: This methodology expands the analytical framework for glycoproteins while retaining the intrinsic advantages of AUC, enabling analysis in native states with high reproducibility as indicated by low standard deviation across replicates. **Conclusions**: The approach was implemented in our proprietary AUC data analysis software called AUCAgent (v1.8.8), providing a new method for glycoprotein quantification and polysaccharide ratio determination in polysaccharide-protein-conjugate vaccines.

## 1. Introduction

Glycosylation modifications of proteins are ubiquitous across all domains of life. These modifications confer a diverse range of functional properties upon proteins [1,2,3] and are a key mechanism for regulating multiple cellular processes, including signal transduction, protein folding, localization, stability, cell–cell interactions, virus-cell recognition, and host immune responses [4,5]. Glycosylation-mediated diversity plays a central role in glycoprotein biosynthesis and biological activity involved in antigen recognition. Notably, viruses exploit certain advantages conferred by glycosylation to assemble their own envelope glycoproteins through host cell glycosylation mechanisms, thereby evading immune surveillance [5,6]. Conversely, biopharmaceutical development has leveraged the ability of glycan structures to specifically recognize various receptors on cell surfaces. Glycosylated therapeutics are increasingly emerging as a favored and valuable therapeutic strategy due to their enhanced targeting specificity and reduced adverse effects as compared to small-molecule drugs [7,8,9].

Many protein drugs possess glycosylation modifications, and the structure of the attached glycan chains significantly affects drug efficacy. The quantity and composition of these glycan chains regulate protein folding, solubility, and intracellular transport processes [10] and result in an extremely broad molecular weight (MW) distribution range in glycoproteins [1]. The average MW of glycan chains directly determines many physicochemical properties, including density, viscosity, diffusivity, sedimentation coefficient, electrophoretic mobility, and specific heat capacity. It also influences protein oligomerization states and the actual mass concentration determined by ultraviolet (UV) absorption methods [1,9,11,12]. These properties influence the biological activity and therapeutic efficacy of glycoprotein drugs. Therefore, the accurate determination of glycoprotein MW has become an indispensable parameter in drug production processes. These measurements are also crucial for comparing batch-to-batch consistency and assessing post-translational modification levels [1,11].

Currently, the determination of protein MW primarily employs techniques such as sodium dodecyl sulfate–polyacrylamide gel electrophoresis (SDS-PAGE), size-exclusion chromatography (SEC), multi-angle light scattering (MALS), analytical ultracentrifugation (AUC), and mass spectrometry (MS). SDS-PAGE is a classic electrophoresis-based method that offers the advantages of simplicity and low cost. However, the accuracy of SDS-PAGE is susceptible to post-translational modifications, such as glycosylation, often leading to MW estimation errors due to migration anomalies [1,13,14]. SEC estimates MW based on protein elution behavior in solution, typically requiring calibration with standards. However, since its response signal depends on the protein’s hydrodynamic radius rather than absolute mass, measurements for non-spherical structures or glycoproteins (where glycan chains significantly increase the hydrodynamic radius) often deviate from the true MW [1,15,16,17]. MALS can directly determine the absolute MW of proteins in solution and effectively assess sample homogeneity without requiring calibration with standards. However, it is typically used with separation techniques such as SEC or AF4 [15]. As an increasingly important analytical tool, AUC is widely regarded as the gold standard technique for evaluating the molecular integrity and non-aggregated state of monoclonal antibodies. AUC can determine MW in solutions approximating natural conditions while simultaneously obtaining multiple other parameters, such as conformation and the sedimentation coefficient [18,19].

However, all the above techniques have certain limitations in determining glycoprotein MW. SDS-PAGE may exhibit MW deviations due to migration abnormalities caused by glycosylation [1,13], and SEC may overestimate actual MW due to the effect of glycan chains on the overall hydrodynamic radius [1,16,17]. Currently, MALS combined with UV detection and differential refractive index (dRI) detection enables the precise MW resolution of both glycan and protein components within glycoprotein complexes, making it a highly accurate method [20]. Conventional AUC detection can utilize dual signals from UV–visible light and interferometric detectors for analysis, a technique already applied in membrane protein complex studies [21]. Furthermore, the AUC analysis software SEDFIT17.0 (https://sedfitsedphat.nibib.nih.gov/software/, accessed on 6 November 2024), in conjunction with the GUSSI glycoprotein analysis module, can also perform glycoprotein analysis provided that the protein’s molecular weight is known in advance. Nevertheless, a single, direct AUC method that can deconvolute protein and glycan mass without requiring a priori knowledge of oligomeric state or theoretical protein mass remains lacking. Consequently, dual-detector AUC analysis holds significant potential for glycoprotein complex characterization.

This study developed a novel absorbance-interference glycoprotein analysis method based on AUC technology. It employs a dual detection system combining UV–visible and interferometric measurements to simultaneously capture sample sedimentation signals. The truncated form of the receptor tyrosine-protein kinase erbB-2(ERBB-2) (UniProt ID: P04626) was employed as a glycosylation pattern protein to analyze the MW and proportion of glycosylated proteins. We integrated SDS-PAGE, SEC, MALS, MP, and LC-MS techniques to cross-validate the algorithm’s reliability. Systematic comparisons were performed to clarify the strengths and limitations of each method in determining glycoprotein MW, demonstrating this approach’s superiority in assessing glycan ratios and MW. The method was integrated into the AUC data analysis software AUCAgent. This study further applied the AUC dual-signal detection strategy to determine the polysaccharide content in polysaccharide-conjugated vaccines. The study provides novel methodological support for analyzing MW or component ratios in biological agents such as glycosylated proteins and polysaccharide-conjugated vaccines.

## 2. Results

### 2.1. SEC and SDS-PAGE Analyses Confirm the High Consistency of Glycoprotein Purification Outcomes

**Figure 1:**
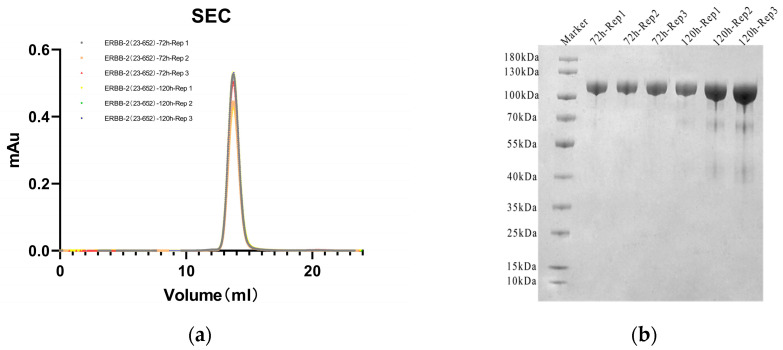
(**a**) Expression of 72 h and 120 h receptor tyrosine-protein kinase (ERBB-2) (23–652) proteins analyzed by SuperdexTM-200 Increase 10/300 GL size-exclusion chromatography. (**b**) Sodium dodecyl sulfate–polyacrylamide gel electrophoresis (SDS-PAGE) results of 72 h and 120 h ERBB-2 (23–652) protein expression.

SEC separates samples through porous stationary phases according to molecular hydrodynamics volume, in which large molecules are eluted first because their size prevents them from entering the smaller pores, and small molecules are eluted later due to their retention by the pores, representing an important means of studying MW and MW distribution. SEC analysis revealed that the retention volumes of ERBB-2 (23–652) proteins expressed in a 293F cell system at 72 and 120 h under the same chromatographic conditions (column model: SuperdexTM-200 Increase 10/300 GL column(Cytiva, Marlborough, MA, USA); mobile phase: phosphate-buffered saline (PBS)) were highly consistent, indicating that the hydrodynamic volume and MW distribution of these glycoproteins in solution were not significantly different (Figure 1a). This phenomenon suggested that the glycoproteins of the proteins with different expression times had similar molecular size characteristics in SEC separation. However, the SEC results for modified proteins, such as glycoproteins, need to be interpreted with caution, as the glycan chains may mask the MW differences in the proteins; thus, these proteins need to be further analyzed in combination with techniques such as MALS.

SDS denatures and uniformly negatively charges proteins, and PAGE separates them by MW, with smaller MW proteins migrating faster. Thus, SDS-PAGE is commonly used to estimate the MW and purity of proteins. In reducing the SDS-PAGE analysis, both the 72 h and 120 h ERBB-2 (23–652) protein samples showed single bands with consistent migration positions, indicating no significant difference in MWs after SDS binding in the unfolded state (Figure 1b). Notably, all the bands migrated at a larger MW position than the theoretical protein MW (71,000 Da), which reflects a migration lag due to glycosylation modification. Thus, the MW of glycoproteins estimated by SDS-PAGE would be overestimated.

### 2.2. MP Characterization of the Consistency of the ERBB-2 (23–652) Protein

MP technology, based on the principle of single-molecule scattering, enables the label-free “weighing” and consistency analysis of molecules in their native state by detecting the intensity of light scattered from biomolecules in a solution under laser illumination; this intensity is proportional to MW [22]. We characterized the homogeneity of the ERBB-2 (23–652) protein samples using MP to validate the SEC measurement results.

**Figure 2:**
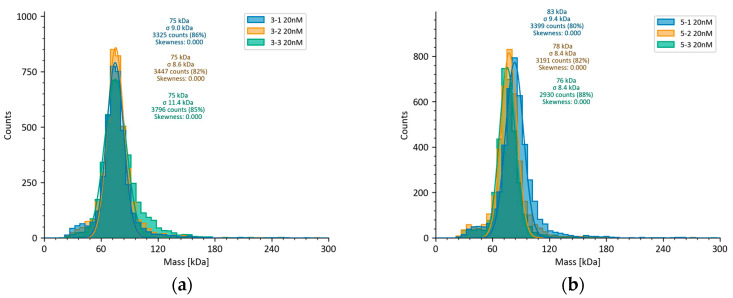
Mass photometry (MP) detection of glycoprotein MW: (**a**) MW measurement of ERBB-2 (23–652) protein using MP at 72 h of expression; (**b**) MW measurement of ERBB-2 (23–652) protein using MP at 120 h of expression.

The MP analysis results indicated a MW of approximately 76,000 Da in the 72 h expression sample, with high uniformity (Figure 2a). In contrast, the 120 h expression sample showed inconsistent results, at 83,000 Da, 78,000 Da, and 76,000 Da, demonstrating lower consistency (Figure 2b). Although the 72 h sample exhibited good uniformity, the MW deviated significantly from the MALS results. The consistency of the 120 h sample also fell below the SEC analysis results. This systematic error is attributed to the MP calibration method, which employed non-glycosylated BSA as a standard. The glycosylation of the actual samples likely alters their scattering properties, rendering the BSA-based calibration inaccurate. In contrast, the absolute measurement techniques MALS and AUC do not require such standards.

### 2.3. LC-MS Analysis of the Average MW of the ERBB-2 (23–652) Protein

**Figure 3:**
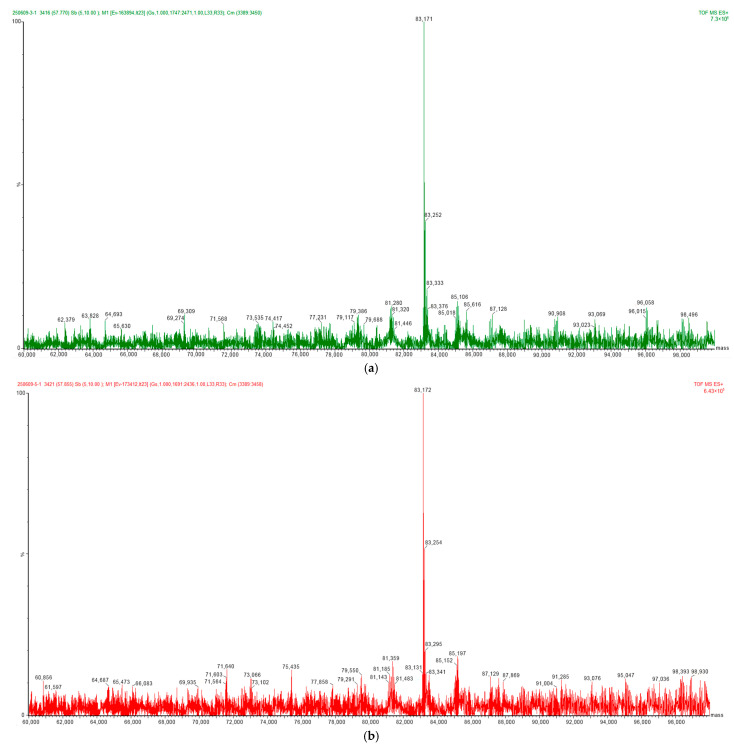
Liquid chromatography–mass spectroscopy (LC-MS) results and deconvoluted spectra of ERBB-2 (23–652) expressed for 72 h and 120 h: (**a**) LC-MS results for ERBB-2 (23–652) protein expressed at 72 h (Rep1). (**b**) LC-MS results for ERBB-2 (23–652) protein expressed at 120 h.

LC-MS, as a mass spectrometry technique capable of high-precision MW determination, is increasingly becoming an important complementary or alternative method in protein analysis and characterization. This study performed LC-MS analysis on the ERBB2-(23–652) proteins expressed at 72 and 120 h. The results showed measured MWs of 83,171 Da and 83,172 Da, respectively (Figure 3). However, complex spectra with low signal-to-noise ratios were observed in all the replicate samples, making effective deconvolution processing using software challenging. This reflects the limitations of the current algorithms when handling such highly glycosylated complex molecules. These findings are consistent with a study by Puranik et al. [11]. reporting that glycosylation interference affected deconvolution in Fc fusion proteins.

### 2.4. SEC-MALS Enables High-Precision Determination of the MW of Intact Glycoproteins

MALS is a technique used to determine the absolute molecular mass of biomolecules. When a macromolecule in solution is irradiated by a beam of laser light, the charge in the molecule is vibrated by the electric field of the light, generating scattered light.

The intensity of the scattered light detected at a fixed angle (*I*(θ) scattered) is directly proportional to the MW of the substance (M), the square of the concentration (c), and the increment of the refractive index increment (*dn*/*dc*), as shown in Equation (1):

(1) $$I{\left(\theta \right)}_{scattered}\propto Mc{\left(\frac{dn}{dc}\right)}^{2}$$

MALS is often used in conjunction with an SEC and a dRI detector to acquire three detector signals simultaneously and characterize the MW and size information of biomolecules. For samples with known *dn*/*dc* or UV extinction coefficients, either UV or dRI data can be selected as the source of the concentration to calculate the MW.

Since scattered light intensity is proportional to the square of the electric field strength ($E^{2}$), the free molecules produce incoherent light with the scattered light intensity proportional to $\left(E1^{2}+E2^{2}\right)$, whereas the bound molecular complexes produce coherent light, with the scattered light intensity proportional to ${\left(E1+E2\right)}^{2}$. Thus, free A/B molecules and bound AB complexes produce different scattering signals, so the overall parameters of the protein complexes need to be calculated by combining UV and dRI using the UV extinction coefficients and *dn*/*dc* of the two substances in the complex. The overall extinction coefficients of the complex and the calculation of *dn*/*dc* are shown in Equations (3) and (4), respectively [20]:

(2) $$C=\frac{A}{\varepsilon_{complex}\times L}=\frac{dRI}{{\left(\frac{dn}{dc}\right)}_{complex}}$$

(3) $${\left(\frac{dn}{dc}\right)}_{complex}={\left(\frac{dn}{dc}\right)}_{protein}\times X_{protein}+{\left(\frac{dn}{dc}\right)}_{modifier}\times \left(1-X_{protein}\right)$$

(4) $$\varepsilon_{compelx}=\varepsilon \times X_{protein}+\varepsilon \times \left(1-X_{protein}\right)$$

(5) $$Mw\propto \frac{I_{LS}}{{\left(\frac{dn}{dc}\right)}_{complex}^{2}\times c}$$

where $X_{protein}$ is the weight fraction of the protein.

**Figure 4:**
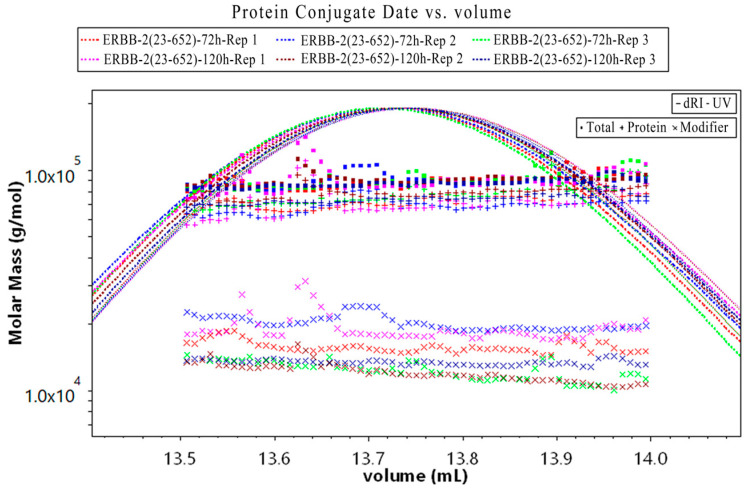
Molecular weight (MW) detected by multi-angle light scattering (MALS). The MW determination of glycoprotein complex components involves two distinct calculations: using protein ultraviolet (UV) extinction coefficients for protein moieties and polysaccharide *dn*/*dc* values for carbohydrate portions. The ■ symbols denote the total molecular mass, + denotes the protein molecular mass, and × denotes the glycan molecular mass.

In light of the above, MALS could be used in conjunction with SEC and a dRI detector to obtain information on the total MW of ERBB-2 (23–652) protein, and the molecular mass of the protein and glycan. The MW distributions of the components of ERBB-2 (23–652) protein expressed for 72 h and 120 h are shown in Figure 4. Their specific MW distributions are shown in Table 1. Some differences in the glycosylation modifications of ERBB-2 (23–652) protein under 72 h and 120 h expression conditions were observed. The polysaccharide percentage (Gly %) of the 72 h samples (Rep1–3) ranged from 13.76% to 22.77%, while the 120 h samples (Rep1–3) ranged from 13.36% to 21.16%, with the overall trend showing slightly less glycosylation in the 120 h samples, but some overlap between the two sets of data. Notably, the polysaccharide content fluctuated widely between the replicate samples within the same time point (e.g., 9.01% difference between Rep1 and Rep3 at 72 h, and 7.8% difference between Rep1 and Rep2 at 120 h), suggesting that the reproducibility of glycoprotein MALS measurements is limited. This variability may arise from glycosylation heterogeneity, where slight site modification differences may alter the light scattering signal and affect the polysaccharide content calculation, even if the degree of glycosylation modification is uniform. Alternatively, it may result from differences in instrument sensitivity in response to the dynamic structure of the glycan chain. Although by this method, SEC-MALS can measure the MW of whole glycoproteins with high accuracy and provide the MWs of both protein and glycan fractions, further validation by other technical means is recommended.

### 2.5. AUC-Based Methods Offer Higher Stability than Other Methods

**Figure 5:**
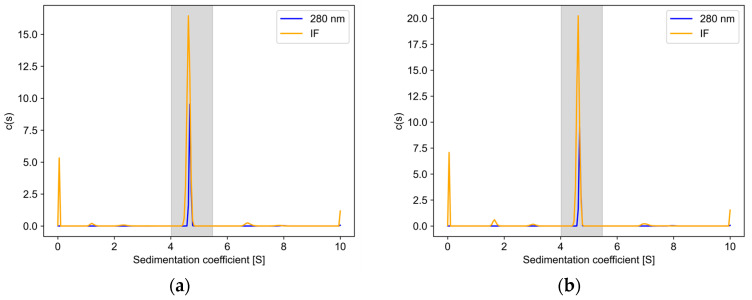
c(*s*) distributions from SEDFIT: (**a**) Sedimentation distribution of ERBB-2 (23–652) protein at 72 h of expression (Rep1). (**b**) Sedimentation distribution of ERBB-2 (23–652) protein at 120 h of expression (Rep1). The shaped area is the glycoprotein integration region in GUSSI and AUCAgent.

Five methods, including LC-MS, SEC-MALS, MP, GUSSI’s, and absorbance-interference method (implemented in AUCAgent), were adopted to analyze glycoproteins. The distribution of the sedimentation coefficient of the ERBB-2 (23–652) protein obtained by AUC is shown in Figure 5.

**Figure 6:**
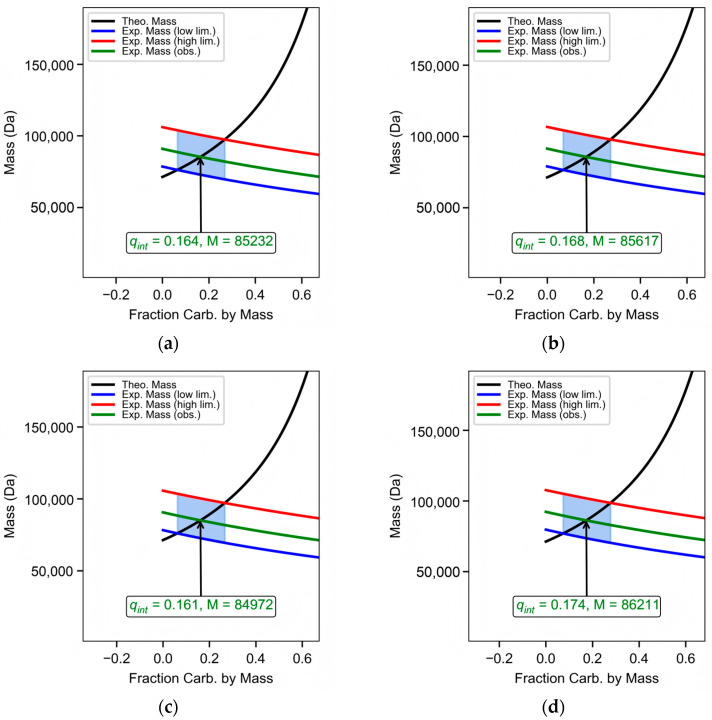

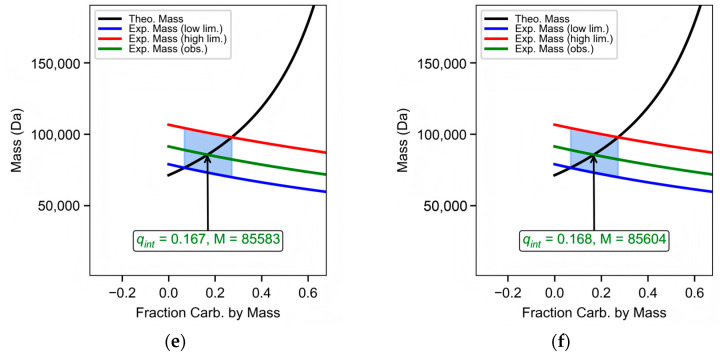
Glycoprotein MW calculated by SEDFIT and GUSSI glycoprotein analysis module: (**a**–**c**) ERBB-2 (23–652) protein MW measurement at 72 h of expression. (**d**–**f**) MW measurement of ERBB-2 (23–652) protein at 120 h of expression.

Among all these methods, the GUSSI glycoprotein analysis module demonstrated superior stability. The average glycan fraction remained stable at about 16.7% in the six groups of experimental data, with a standard deviation of 0.4382 (Figure 6 and Table 2). The absorbance-interference method ranked second in reliability. The average glycan fraction calculated by this method was 12.73%, with a standard deviation of 0.7284 (Table 3). In comparison to the two preceding AUC-based methods, SEC-MALS exhibited relatively lower stability, with an average value of 17.38% and a standard deviation of 3.9196. Further statistical analysis of MALS and the two AUC-based methods (the GUSSI glycoprotein analysis module and the absorbance-interference method) across the two expression groups (Table S1, Figure S1) revealed that the absorbance-interference method exhibited the lowest dispersion in the 120 h sample group (with the smallest standard deviation of 0.0027), indicating the highest stability within that set. Overall, the GUSSI glycoprotein analysis module demonstrated the highest stability (standard deviation: 0.0036), followed by the absorbance-interference method (0.0067), while MALS showed the poorest performance (0.174). A two-way ANOVA test was conducted, and the results (Table S2) indicate that there was no significant difference between the 72 h and 120 h sample groups (*p* = 0.9409 > 0.05). However, significant differences were observed among the different detection methods. Subsequent Tukey HSD (Honestly Significant Difference) analysis further showed that the absorbance-interference method differs significantly from the other two methods (Table S3).

In addition, we observed discrepancies in MW and glycan content among MALS, MP, the GUSSI glycoprotein analysis module, and the absorbance-interference method (Table 4). These differences are expected and primarily arise from the distinct principles and assumptions underlying each technique. MALS relies on laser light scattering and concentration signals (from UV or dRI detectors) for MW determination. Its accuracy is influenced by laser noise, requiring optimal instrument conditions. Furthermore, accurate concentration determination for macromolecular complexes such as glycoproteins requires the integration of signals from both UV and dRI detectors. MP technology depends on calibration against standards, making its results susceptible to differences between the standards and the actual sample properties. The GUSSI glycoprotein analysis module requires the theoretical MW of the protein as prior information to calculate the total MW; therefore, the accuracy of the theoretical MW setting is crucial for its final MW and glycan content results. The absorbance-interference method works by comparing concentration differences between two measurement signals to directly obtain the ratio of glycans and proteins in glycoproteins. For reliable results, on the one hand, it is crucial to have precise calibration between the signals and the corresponding concentration values. Thus, two crucial optical properties, the protein extinction coefficient and the refractive index increments of both proteins and glycans in glycoproteins, must be considered when using this method. However, the accuracy of MW measurement depends on the accuracy of the frictional ratio ($f/f_{0}$) fitting by c(s) sedimentation coefficient distribution analysis in SEDFIT. More accurate $f/f_{0}$ determinations yield more reliable MW measurements.

### 2.6. AUC Can Accurately Determine the Polysaccharide Ratio in Polysaccharide-Conjugated Vaccines

Polysaccharide-conjugated vaccines represent a novel vaccine type created by chemically linking bacterial capsular polysaccharides to protein carriers via covalent bonds. They effectively stimulate T-cell-dependent immune responses, overcoming the limitations of conventional vaccines, such as weak immunogenicity in infants and young children and an inability to induce lasting immune memory. This significantly enhances the protective efficacy and induces long-term immunity. Currently, this type of vaccine has been successfully applied to prevent infections caused by multiple pathogens, including *Hemophilus influenzae* type b, *Streptococcus pneumoniae*, and *Neisseria meningitidis* [18,23,24]. Although different polysaccharide-conjugated vaccines vary in their specific conjugation processes and typically involve multi-step reactions, their polysaccharide ratios are critical quality control indicators. These factors directly impact the stability, safety, efficacy, and batch-to-batch consistency of the conjugates. Therefore, this study applied the aforementioned algorithm to further explore its ability to determine polysaccharide ratios in polysaccharide-conjugated vaccines (Table 5).

**Figure 7:**
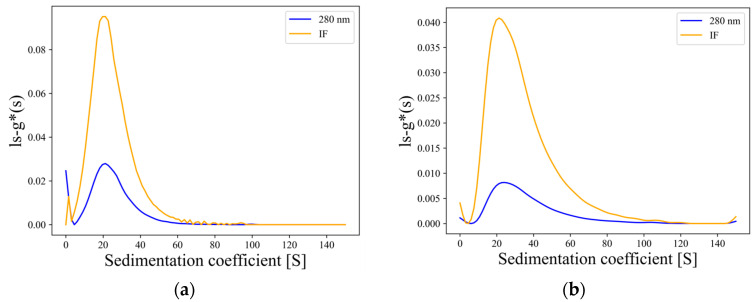
ls-g*(s) distributions from AUCAgent: (**a**) Sedimentation distribution of polysaccharide-conjugated vaccine-1 (Rep1). (**b**) Sedimentation distribution of polysaccharide-conjugated vaccine-2 (Rep1).

Both vaccines utilize tetanus toxoid as the carrier protein but exhibit significant differences in protein content. We measured sedimentation velocity in the vaccine samples using AUC, simultaneously collecting absorbance values at 280 nm and interference light signals. The sedimentation distribution results are shown in Figure 7. The polysaccharide ratios in the samples were obtained through algorithmic analysis of the data. The summary results are presented in Table 6.

Both vaccines exhibited broad sedimentation coefficient distributions (Figure 7), consistent with the broad MW distribution observed by Jia et al. [25] for polysaccharide-conjugated vaccines using MALS technology. The results reflect significant compositional heterogeneity within the samples in solution. Therefore, based on the difference between the 280 nm and interference light dual signals, we selected the entire sedimentation distribution region and used the absorbance-interference method to calculate polysaccharide ratios. Each vaccine was tested in triplicate, and the results showed good consistency between replicates. The reproducible performance of AUCAgent highlights its potential for assessing vaccine batch-to-batch consistency and for providing orthogonal verification between different analytical methods in component distribution analysis.

## 3. Discussion

The GUSSI glycoprotein analysis module balances simplicity and robustness by fixing the protein’s MW to its theoretical value. However, in practice, experimentally observed apparent MW often deviates from theoretical values. Enforcing strict MW constraints could systematically skew results, particularly for glycoproteins with heterogeneous glycosylation. Precisely because of this molecular weight constraint, which acts as a regularization on the glycoprotein calculation results, it yields solutions with greater stability. In contrast, the absorbance-interference method lacks a similar constraint, which accounts for the difference in stability between the two methods. Another essential factor in the GUSSI glycoprotein analysis module is the oligomeric state of the protein. This is difficult to estimate experimentally, even if the actual protein monomer sequence is known. The absorbance-interference method entirely bypasses the need to account for protein multimerization. It relies solely on the protein’s molar extinction coefficient, the *dn*/*dc* of the protein, and the *dn*/*dc* of the glycan to accurately quantify glycan content. If the protein sequence is known, its extinction coefficient and *dn*/*dc* value can be computationally predicted. For glycans, *dn*/*dc* values show minimal variation across types (e.g., 0.142–0.150 mL/g), allowing the use of a generic value when experimental data is unavailable. Building on these parameters, this method enables the straightforward quantification of glycan content in glycoproteins, overcoming the limitations of the GUSSI glycoprotein analysis module requiring knowledge of the multimerization state. Predicted or generic parameters may not precisely match sample-specific values, but deviations from true values are typically minor. Furthermore, our findings demonstrate that the combined application of the absorbance-interference method and the GUSSI glycoprotein analysis module enables the estimation of glycoprotein oligomeric states.

While the SEC analysis cannot provide detailed glycan composition data, it confirmed batch-to-batch consistency across the six sample sets. MALS shares a similar fundamental principle with the AUC-based absorbance-interference method, given that both methods rely on the integration of two detection modalities for molecular characterization. However, MALS exhibits significantly higher variability compared to the AUC method, which underscores the superior reproducibility and precision of AUC in glycoprotein analysis. While the current results support the feasibility of this method, certain limitations of the absorbance-interference method adopted in AUCAgent should be considered. First, due to the structural complexity of glycan modifications, the dn/dc values may vary among different glycans. The applicability of the commonly used generic dn/dc parameters for more intricately modified glycan structures still requires further experimental validation. Second, the calculation relies on the extinction coefficient of the protein, yet the influence of glycosylation on this parameter remains unclear. Therefore, the potential effects of these two aspects should be carefully considered during data interpretation.

Furthermore, the analysis of the polysaccharide-conjugated vaccines showed that the absorbance interference method could directly quantify polysaccharide ratios. Given the common polydispersity of such vaccines, the absorbance interference strategy employed by AUCAgent not only offers superior reproducibility and precision in glycoprotein quantification compared to other methods but also demonstrates significant advantages in analyzing ratios in polysaccharide-conjugated vaccines, indicating substantial application potential.

## 4. Materials and Methods

### 4.1. Protein Expression and Purification

The receptor tyrosine-protein kinase erbB-2 (ERBB-2) (23–652) gene sequence was synthesized by Ruibiotech. The truncated protein contained seven N-glycosylation sites located at positions 68, 125, 187, 259, 530, 571, and 629 of the sequence. These were amplified by PCR after cloning into a pVAX1 vector, and a 6 × His-tagged fusion protein was generated, and the ERBB-2 (23–652) protein was expressed in a 293F cell system. The 293F cells were cultured to a cell concentration of 2 × 10^6^ cells/mL, and the mass ratio of plasmid to PEI was about 1:4 for transfection, and 1 mg of plasmid per liter of cells. The cells were transfected under the same conditions, and the cellular supernatants were collected at 72 and 120 h. Three sets of replicates were constructed at the two time points, with 1 L of cells for each set of replicates.

The cell supernatant was collected and then subjected to PBS (pH 7.4) buffer replacement using a 10,000 Da small ultrafiltration membrane pack (Merck, P2C100C01) via an ultrafiltration concentration system (Merck Millipore Mini Pellicon, Merck, Darmstadt, Germany). The concentrated protein solution was subjected to affinity chromatography using a His affinity column (Cytiva, Marlborough, MA, USA, 17531802). Heterogeneous proteins were removed using wash buffer (PBS, 20 mM imidazole, pH 7.4), and target proteins were eluted using elution buffer (PBS, 400 mM imidazole). The eluted proteins were concentrated using a 10,000 Da centrifugal filter (Merck, UFC905008) and further purified using a SuperdexTM-200 Increase 5/150 GL column (Cytiva, 28990945) equilibrated with PBS buffer. The proteins were finally stored in PBS buffer.

### 4.2. Polysaccharide-Conjugated Vaccine Manufacturing Process

The Streptococcus pneumoniae serotypes (22F using the amine reduction method and 19A using the 1-cyano-4-dimethylaminopyridinium tetrafluoroborate (CDAP) method) were conjugated to the protein of diphtheria toxoid as the carrier to form covalently bound polysaccharide–protein conjugates. The purification was carried out by tangential flow ultrafiltration and molecular sieve chromatography. The glycan content was determined according to the anthrone-sulfuric acid method in the current edition of the *Chinese Pharmacopoeia* (Part III).

### 4.3. Size-Exclusion Chromatography

An Agilent 1260 infinity liquid chromatography system, equipped with an autosampler, a thermostatic column chamber, a UV detector, and a temperature control at 20 °C, was used for chromatographic separation. A SuperdexTM-200 Increase 10/300 GL column (Cytiva, 28990944) was used with PBS as the mobile phase and a detector wavelength of 280 nm. The protein concentration of ERBB-2 (23–652) purified at 72 h and 120 h was quantified uniformly to an A280 nm value of about 0.6. In total, 100 µL of each sample was uploaded, the run flow rate was 0.5 mL/min, and the run time was 48 min.

### 4.4. Sodium Dodecyl Sulfate–Polyacrylamide Gel Electrophoresis

Genscript YoungPAGE™ Protein Prep Gel (Genscript, Nanjing, China, M00928) was used for the electrophoretic separation of the samples. The protein samples were mixed with protein loading buffer (SDS-PAGE sample loading buffer, 5× (Beyotime, Shanghai, China, P0015)) at a volume ratio of 4:1 prior to sampling and denatured at 100 °C for 5 min, after which gel electrophoresis was performed. Pre-stained protein marker (Thermo Scientific, Waltham, MA, USA, 26616), containing 10 MW markers, 180, 130, 100, 70, 55, 40, 35, 25, 15, and 10,000 Da protein bands, was used for protein MW estimation. Staining was performed using the eStain^®^ L1 Protein Stainer (Genscript, Nanjing, China) after completing electrophoresis.

### 4.5. Liquid Chromatography–Mass Spectroscopy

In the LC-MS analysis, the analytes were separated by a 60 min gradient elution at a flow rate of 0.5 µL/min with a nanoACQUITY UPLC system, which was directly interfaced with a SYNAPT-G2-Si mass spectrometer produced by the Waters company (Milford, MA, USA). The analytical column was a Protein BEH C4 silica capillary column (150 µm ID, 100 mm long) packed with C-4 resin (300 Å, 1.7 µm) purchased from the Waters company. Mobile phase A consisted of 0.1% formic acid aqueous solution, and mobile phase B consisted of 100% acetonitrile and 0.1% formic acid.

### 4.6. Multi-Angle Light Scattering

An Agilent 1260 infinity liquid chromatography system configured with an autosampler, a thermostat column chamber, a UV detector, and temperature control at 20 °C was used for MALS analysis. Chromatographic separation was achieved using a SuperdexTM-200 Increase 10/300 GL column (Cytiva, 28990944) connected to a Wyatt DAWN HELEOS laser detector (Wyatt, Santa Barbara, CA, USA) and a Wyatt Optilab T-rEX differential detector (Wyatt, CA, USA). A PBS mobile phase overnight equilibration system and a detector were used. The 72 h and 120 h purified ERBB-2 (23–652) protein concentrations were quantified to an A280 nm value of about 0.6, and 100 µL of the samples were uploaded. Light scattering, UV, and differential signals were collected, and the data were analyzed using ASTRA 8.1.2 software.

### 4.7. Mass Photometry

The solution-phase mass determination of native glycoproteins was acquired using a TwoMP (Refeyn, Ltd., Oxford, UK) mass photometer calibrated with BSA (66,000 Da). Experimental data were obtained in the form of mass photometry movies acquired over 6000 frames (60 s) using the AcquireMP software (2024R2) on precleaned, high-sensitivity microscope slides [n]. The test samples (200 nM) were mixed with 18 µL of PBS to a final concentration of 20 nM, and then loaded onto a glass slide for measurement. The final protein concentrations were determined empirically to achieve approximately 50 binding events per second.

### 4.8. Analytical Ultracentrifugation

AUC (Optima AUC, BeckmanCoulter, Brea, CA, USA) sedimentation velocity experiments were performed using both absorbance (280 nm) and interference (660 nm) optical detection systems. The rotor speed was maintained at 45,000 rpm throughout the sedimentation process. Real-time scanning data were acquired at 120 s intervals, with absorbance recorded at 280 nm (for protein detection) and interference signals monitored at 660 nm (for high-precision concentration profiling).

The collected data were processed using SEDFIT software with the fitting parameters set to 200 for resolution, 0 for s_min_, 10 for s_max_, and a confidence level of 0.68 to obtain the results of the continuous c(s) distribution of the 280 nm and interferometric data for each group of samples. Both distributions were imported into GUSSI 2.1.0 software and analyzed for glycoproteins using the Glycoprotein Calcs module, selecting a sedimentation coefficient range of 4.0–5.5 S. The protein fitting parameters were set to a UV extinction coefficient of 0.9 $L\cdot g^{-1}\cdot cm^{-1}$, and the protein MW was set to 71,250 Da (the above parameters were predicted from https://www.expasy.org/ based on the protein amino acid sequence). The corresponding $f/f_{0}$ values were entered as distribution parameters, which were obtained by fitting with the SEDFIT software to obtain the final glycoprotein mass distribution results.

In the sedimentation velocity experiments, the particle movement process in solution can be described by the Lamm equation:

(6) $$\frac{\partial c}{\partial t}=\frac{1}{r}\frac{\partial }{\partial r}(rD\frac{\partial c}{\partial r}-{s\omega }^{2}r^{2}c)$$

where c, t, r, D, ω, and s represents the concentration of the solute, sedimentation time, radial distance from the axis of rotation, diffusion coefficient, angular velocity of the rotor, and sedimentation coefficient, respectively. The sedimentation coefficient is defined as

(7) $$s=\frac{u}{\omega^{2}r}$$

and u is a measure of the sedimentation speed of macromolecules when using the centrifugation method, which is equal to the speed per unit of the centrifugal field. The sedimentation coefficient unit is Svedberg (symbol S), which is a unit of time and is equal to $10^{-13}$ s. The diffusion coefficient of a molecule is related to its translational frictional coefficient, f, through the Einstein–Smoluchowski relationship:

(8) $$D=\frac{RT}{N_{0}f}=\frac{k_{B}}{f}$$

The unit of diffusion coefficient is $cm^{2}/s$, also called Fick’s (symbol F), where 1 F = $10^{-7}cm^{2}/s$.

### 4.9. GUSSI Glycoprotein Calculations

GUSSI (Version 2.1.0) includes a glycoprotein analysis module that efficiently calculates glycan composition ratios. It uses c(s) distributions from SEDFIT, combined with partial specific volumes, experimental temperature, the experimental solution density, viscosity, and $f/f_{0}$, to directly quantify the fraction of the total mass of the carbohydrate in glycoprotein complexes. GUSSI’s theory for calculating glycoproteins is as follows: First, the assumed glycan fraction is q, and the hypothetical total glycoprotein molar mass is $M_{GP,h}$:

(9) $$M_{GP,h}=\frac{nM_{p,mono}}{1-q}$$

The apparent molecular mass $M_{GP,s}$ can also be calculated from the experimental data according to the Svedberg equation:

(10) $$M_{GP,s}=\frac{s_{GP}RT}{D_{GP}(1-{\bar{v}}_{GP}\rho )}$$

where $s_{GP}$ is the sedimentation coefficient of glycoprotein. The $\bar{v}$ of glycoprotein needs to be recalculated based on the glycan fraction:

(11) $${\bar{v}}_{GP}=q\bar{v}c+\left(1-q\right){\bar{v}}_{P}$$

It is easy to calculate the value of q, so that the hypothetical molar mass and the apparent molecular mass are consistent:

(12) $$q=\frac{1-\left[\psi \left(1-{\bar{v}}_{P}\right)\right]}{1+\left[\psi \rho \left({\bar{v}}_{P}-{\bar{v}}_{C}\right)\right]}$$

where

(13) $$\psi =\frac{nM_{P,mono}D_{GP}}{s_{GP}RT}$$

The sedimentation distributions used in GUSSI are all taken from absorbance-type data, because the $f/f_{0}$ is affected by all species, as more species can be detected in the interference optics.

### 4.10. AUCAgent Glycoprotein Calculations

AUCAgent is a professional software developed by Wenqi Li’s team for processing AUC data. It currently includes three analytical models: g(*s*), ls-g(*s*), and c(*s*), and integrates the glycoprotein analysis module described in this chapter. The software is freely available and can be downloaded from https://www.frcbs.tsinghua.edu.cn/download/software/133 (accessed on 14 August 2024).

Unlike GUSSI, the analysis of glycoprotein by AUCAgent is based on absorbance optics and interference optics, namely the absorption-interference difference method. In the interference optical system, the number of fringes J follows [26]:

(14) $$J=c\frac{dn}{dc}\frac{l}{\lambda }$$

where c is the concentration, and dn/dc is the specific refractive increment. We used 0.185 mL/g for proteins and 0.15 mL/g for glycans. l is the optical pathlength, and λ is the wavelength of the light used. In the absorbance optical system, the measured absorbance follows the Beer–Lambert law:

(15) A=εlc

where ε is the molar extinction coefficient per cm, c is the concentration, and l is the optical pathlength determined by the centerpiece type.

The quantitative determination of glycan occupancy in glycoprotein involves three sequential calculations: total glycoprotein ($c_{GP}$) concentration is calculated using Equation (16):

(16) $$c_{\mathrm{GP}}=\frac{J\lambda }{l\cdot \frac{dn}{dc}}$$

protein core concentration ($c_{P}$) is calculated using Equation (17):

(17) $$c_{P}=\frac{A}{l\cdot \varepsilon }$$

and the glycan ratio is computed using $c_{GP}$ and $c_{P}$:

(18) $$q=\frac{c_{GP}-c_{P}}{c_{GP}}.$$

As mentioned before, $\bar{v}$ needs to be recalculated (Equation (11)) before calculating the glycoprotein total mass. The corresponding glycan proportion can be calculated using the process described above under the relevant parameters.

## Figures and Tables

**Table 1:** Results of molecular weight (MW) and glycan content calculated by multi-angle light scattering (MALS).

Sample ID	Total MW (Da)	Protein MW (Da)	Gly MW (Da)	Gly %
ERBB2-(23–652)-72h-Rep1	88,880 (±5.081%)	73,020 (±5.075%)	15,860 (±5.107%)	17.84
ERBB2-(23–652)-72h-Rep2	89,630 (±4.388%)	69,220 (±4.385%)	20,410 (±4.4%)	22.77
ERBB2-(23–652)-72h-Rep3	90,610 (±5.82%)	78,140 (±5.83%)	12,470 (±5.579%)	13.76
ERBB2-(23–652)-120h-Rep1	91,510 (±6.187%)	72,150 (±6.153%)	19,360 (±6.317%)	21.16
ERBB2-(23–652)-120h-Rep2	91,030 (±5.73%)	78,870 (±5.723%)	12,160 (±5.778%)	13.36
ERBB2-(23–652)-120h-Rep3	87,600 (±1.622%)	74,120 (±1.625%)	13,470 (±1.614%)	15.38
Average				17.38
Std				3.9196

**Table 2:** Results of MW and glycan content calculated by GUSSI.

Sample ID	Total MW (Da)	Protein MW (Da)	Gly MW (Da)	Gly %
ERBB2-(23–652)-72h-Rep1	85,232	71,254	13,978	16.4
ERBB2-(23–652)-72h-Rep2	85,617	71,233	14,384	16.8
ERBB2-(23–652)-72h-Rep3	84,972	71,292	13,680	16.1
ERBB2-(23–652)-120h-Rep1	86,211	71,210	15,001	17.4
ERBB2-(23–652)-120h-Rep2	85,583	71,291	14,292	16.7
ERBB2-(23–652)-120h-Rep3	85,604	71,223	14,381	16.8
Average				16.7
Std				0.4382

**Table 3:** Results of MW and glycan content calculations by the absorbance-interference (implemented in AUCAgent).

Sample ID	Total MW (Da)	Protein MW (Da)	Gly MW (Da)	Gly %
ERBB2-(23–652)-72h-Rep1	86,792	76,646	10,146	11.69
ERBB2-(23–652)-72h-Rep2	87,128	76,507	10,621	12.19
ERBB2-(23–652)-72h-Rep3	86,173	75,393	10,780	12.51
ERBB2-(23–652)-120h-Rep1	87,630	76,159	11,471	13.09
ERBB2-(23–652)-120h-Rep2	86,731	75,222	11,509	13.27
ERBB2-(23–652)-120h-Rep3	86,632	74,824	11,808	13.63
Average	-	-	-	12.73
Std				0.7284

**Table 4:** Mass spectroscopy (MS), MALS, mass photometry (MP), and analytical ultracentrifugation (AUC) results for calculating total protein MW and glycan content.

Sample ID	MS MW Total (Da)	MALS MW Total (Da)	MP MW Total (Da)	AUC-SEDFIT-280 MW Total (Da)	AUC-SEDFIT-IF MW Total (Da)	GUSSI MW Total (Da)	MS Gly %	MALS Gly %	GUSSI Gly %
ERBB2-(23–652)-72h-Rep1	83,171	88,880 (±5.081%)	75,000	91,555	97,525	85,232	14.33%	17.80%	16.40%
ERBB2-(23–652)-72h-Rep2	/	89,630 (±4.388%)	75,000	92,087	95,735	85,617	/	22.80%	16.80%
ERBB2-(23–652)-72h-Rep3	/	90,610 (±5.82%)	75,000	91,189	94,251	84,972	/	13.80%	16.10%
ERBB2-(23–652)-120h-Rep1	83,172	91,510 (±6.187%)	83,000	92,938	95,444	86,211	/	21.20%	17.40%
ERBB2-(23–652)-120h-Rep2	/	91,030 (±5.73%)	78,000	92,047	93,917	85,583	14.33%	13.40%	16.70%
ERBB2-(23–652)-120h-Rep3	/	87,600 (±1.622%)	76,000	92,070	94,257	85,604	/	15.40%	16.80%

**Table 5:** Polysaccharide-conjugated vaccine information.

Sample ID	Buffer	Polysaccharide Ratio (%)
Polysaccharide-conjugated vaccine-1	0.85% NaCl	37.1
polysaccharide-conjugated vaccine-2	0.85% NaCl	57.6

**Table 6:** AUC results were used to calculate the polysaccharide ratio of polysaccharide-conjugated vaccines.

Sample ID	Buffer	Polysaccharide Ratio (%)
Polysaccharide-conjugated vaccine-1-Rep1	0.85% NaCl	40.9
Polysaccharide-conjugated vaccine-1-Rep2	0.85% NaCl	41.1
Polysaccharide-conjugated vaccine-1-Rep3	0.85% NaCl	40.01
Polysaccharide-conjugated vaccine-2-Rep1	0.85% NaCl	57.1
Polysaccharide-conjugated vaccine-2-Rep2	0.85% NaCl	59.7
Polysaccharide-conjugated vaccine-2-Rep3	0.85% NaCl	60.2

## Data Availability

The original contributions presented in this study are included in the article/Supplementary Material. Further inquiries can be directed to the corresponding authors.

## Supplementary Materials

The following supporting information can be downloaded at: https://www.mdpi.com/article/10.3390/ph19020210/s1, Table S1: Descriptive Statistics Table (ERBB2-(23-652)). Table S2: Two-way ANOVA Summary Table (ERBB2-(23-652)). Table S3: Two-way ANOVA Summary Table (ERBB2-(23-652)). Figure S1. The mean interaction graphs and box plots of different expression times (a) and detection methods (b).
