# Supplementary material for: Quantification of Glycan in Glycoproteins via AUCAgent-Enhanced Analytical Ultracentrifugation
Source: Pharmaceuticals (Basel). 2026 Jan 26;19(2):210. doi: 10.3390/ph19020210 (PMC12943701; doi:10.3390/ph19020210)
Supplement: Supplementary file 1 [file pharmaceuticals-19-00210-s001.zip › pharmaceuticals-4077734-supplementary.pdf]

Supplementary Material

Table S1. Descriptive Statistics Table (ERBB2-(23-652))

Method	Expression Time	Sample Size (n)	Mean	Standard Deviation (SD)	Range (Max-Min)
AUCAgent	72h	3	0.1213	0.0041	0.0082
	120h	3	0.1333	0.0027	0.0054
	Combined	6	0.1273	0.0067	0.0194
GUSSI	72h	3	0.1643	0.0035	0.007
	120h	3	0.1697	0.0038	0.007
	Combined	6	0.167	0.0036	0.013
MALS	72h	3	0.1813	0.0451	0.09
	120h	3	0.1667	0.0405	0.078
	Combined	6	0.174	0.0415	0.094

Table S2. Two-way ANOVA Summary Table (ERBB2-(23-652))

Source of Variation	df	Sum of Squares	F-value	p-value
Expression Time (Group)	1	0.000004	0.005726	0.94093
Detection method (Method)	2	0.007612	6.128836	0.014655
Group × Method interaction	2	0.000578	0.465203	0.638873

Table S3. Two-way ANOVA Summary Table (ERBB2-(23-652))

Comparison	Mean Difference	Std. Error	95% Confidence Interval	Adjusted p-value
AUCAgent vs GUSSI	-0.0397	0.0114	(-0.0672, -0.0122)	0.0078*
AUCAgent vs MALS	-0.0467	0.0114	(-0.0742, -0.0192)	0.0020**
GUSSI vs MALS	-0.007	0.0114	(-0.0345, 0.0205)	0.8475

*p < 0.05; **p < 0.01*

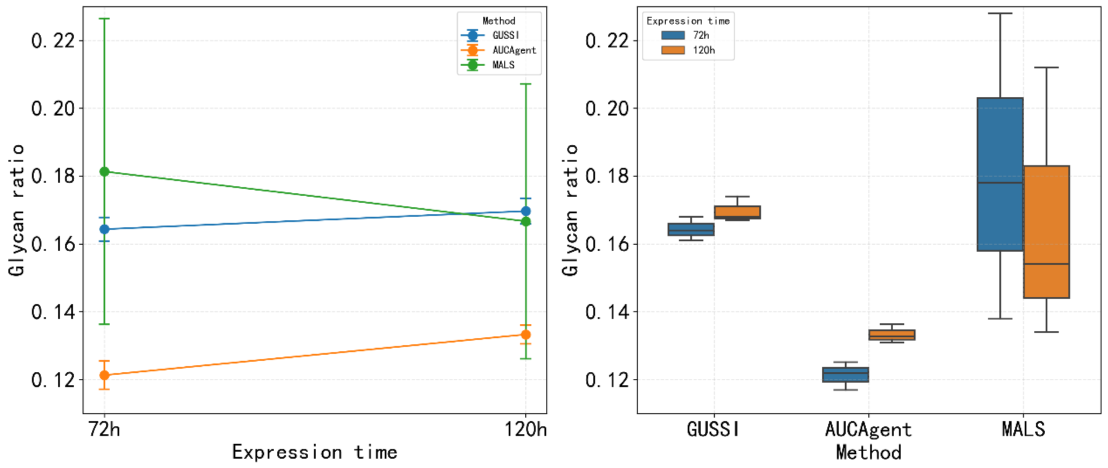

(a) (b)

Figure S1. The mean interaction graphs and box plots of different expression times (a) and detection methods (b).
